# Maladaptive Changes Associated With Cardiac Aging Are Sex-Specific and Graded by Frailty and Inflammation in C57BL/6 Mice

**DOI:** 10.1093/gerona/glaa212

**Published:** 2020-08-28

**Authors:** Alice E Kane, Elise S Bisset, Stefan Heinze-Milne, Kaitlyn M Keller, Scott A Grandy, Susan E Howlett

**Affiliations:** 1 Department of Genetics, Harvard Medical School, Boston, Massachusetts; 2 Charles Perkins Center, The University of Sydney, Australia; 3 Department of Pharmacology, Dalhousie University, Halifax, Nova Scotia, Canada; 4 School of Health and Human Performance, Dalhousie University, Halifax, Nova Scotia, Canada; 5 Department of Medicine (Geriatric Medicine), Dalhousie University, Halifax, Nova Scotia, Canada

**Keywords:** Chemokines, Echocardiography, Frailty index, Pro-inflammatory cytokines, Sex differences

## Abstract

We investigated whether late-life changes in cardiac structure and function were related to high levels of frailty and inflammation in male and female mice. Frailty (frailty index), ventricular structure/function (echocardiography), and serum cytokines (multiplex immunoassay) were measured in 16- and 23-month-old mice. Left ventricular (LV) mass and septal wall thickness increased with age in both sexes. Ejection fraction increased with age in males (60.4 ± 1.4 vs 68.9 ± 1.8%; *p* < .05) but not females (58.8 ± 2.5 vs 62.6 ± 2.4%). E/A ratios declined with age in males (1.6 ± 0.1 vs 1.3 ± 0.1; *p* < .05) but not females (1.4 ± 0.1 vs 1.3 ± 0.1) and this was accompanied by increased ventricular collagen levels in males. These changes in ejection fraction (*r* = 0.52; *p* = .01), septal wall thickness (*r* = 0.59; *p* = .002), E/A ratios (*r* = −0.49; *p* = .04), and fibrosis (*r* = 0.82; *p* = .002) were closely graded by frailty scores in males. Only septal wall thickness and LV mass increased with frailty in females. Serum cytokines changed modestly with age in both sexes. Nonetheless, in males, E/A ratios, LV mass, LV posterior wall thickness, and septal wall thickness increased as serum cytokines increased (eg, IL-6, IL-3, IL-1α, IL-1β, tumor necrosis factor-α, eotaxin, and macrophage inflammatory protein-1α), while ejection fraction declined with increasing IL-3 and granulocyte-macrophage colony stimulating factor. Cardiac outcomes were not correlated with inflammatory cytokines in females. Thus, changes in cardiac structure and function in late life are closely graded by both frailty and markers of inflammation, but this occurs primarily in males. This suggests poor overall health and inflammation drive maladaptive changes in older male hearts, while older females may be resistant to these adverse effects of frailty.

Cardiovascular diseases such as heart failure increase with age in both men and women (1). The reasons for this are unclear and likely multifactorial. One contributing factor could be adverse structural and functional remodeling in the aging heart, which occurs in the absence of overt cardiovascular disease (2,3). This remodeling may promote the development of diseases like heart failure in later life (2,3). Emerging data from human and animal studies suggests that the impact of age on the heart differs between the sexes (2–4). This suggests that cardiac aging may be sex-specific, although the mechanisms are poorly understood. Understanding the influence of age and sex on the heart is important as this may help explain why men and women are predisposed toward different cardiovascular diseases as they age (5).

Although chronological age can adversely affect the heart, aging is heterogeneous and not all individuals age at the same rate (6). The notion of frailty was introduced by demographers in 1979 to describe heterogeneity in the risk of death in people of the same age (7). While there is no internationally established definition of frailty, this term is now used to describe a state of high vulnerability to adverse health outcomes in individuals of the same age (8). Clinical studies have shown that frailty increases the risk of heart failure in later life, although why this occurs is unclear (9,10). As chronic inflammation is implicated in both frailty (11) and heart failure (9), it is possible that chronic inflammation promotes maladaptive cardiac remodeling that sets the stage for heart failure in frail individuals.

A key challenge to understanding heart disease in the older frail men and women who develop it, is the use of appropriate models (12,13). Most basic research studies on cardiovascular aging use only male animals, and few have considered frailty (14,15). We pioneered the idea that frailty can be measured in mice with a “frailty index” (FI) based on accumulation of age-related health deficits (16,17), parallel to the FI approach used clinically (18). We showed that female mice have higher frailty scores than males (19), as seen in people (20,21). Our work also shows that mice with high frailty scores have high serum levels of pro-inflammatory cytokines, indicative of chronic inflammation (19,22).

In this study, we hypothesized that adverse changes in ventricular structure and function would be greatest in mice with high levels of frailty and inflammation. Our objectives were: (i) to evaluate late-life changes in ventricular structure and function in aging mice; (ii) to investigate whether these changes were the most prominent in mice with high frailty scores; (iii) to assess the relationship between adverse changes in the heart and markers of inflammation; and (iv) to determine if relationships between frailty, inflammation, and cardiac aging differed between the sexes. We used echocardiography to follow C57BL/6 mice of both sexes from middle age to later life. Frailty was assessed with an FI and serum cytokines were measured with a multiplex assay. Our results show that alterations in heart structure and function in later life are strongly predicted by both individual health status and inflammation, especially in male animals.

## Method

### Animals

Three-week-old male and female C57BL/6 mice were purchased from Charles River (St. Constant, Quebec, Canada). Mice were housed in groups of 3 to 5 littermates per cage (Individually Ventilated Caging System, Allentown Inc., Allentown, NJ) and raised in the Dalhousie University Carlton Animal Care Facility for up to 16 months before experiments were initiated. This is a clean, multispecies facility dedicated to housing-specific pathogen-free mice and rats. Mice were maintained at 21°C with 35% humidity. Animals experienced a 12-hour light–dark cycle with ad libitum access to food (Prolab RMH3000, LabDiet, St. Louis, MO) and water. Animal husbandry duties were performed in animal transfer stations. Our studies were approved by the Dalhousie University Committee on Laboratory Animals and conformed to the Canadian Council on Animal Care guidelines. Frailty scores for some mice in the present study (*n* = 21) were included in a previous publication (23) where they were examined with respect to different outcomes (eg, myocyte stimulation; quantification of myofilament proteins). Frailty scores for most mice and the outcomes measured here have not been previously reported.

### FI Assessment

Frailty assessments used the 31-item mouse clinical FI instrument described in detail elsewhere (17). In brief, mice were acclimated in a quiet room in the animal care facility. Each mouse was assessed clinically for age-associated health deficits across a wide range of systems (eg, integument, musculoskeletal, vestibulocochlear/auditory, ocular/nasal, digestive, urogenital and respiratory systems, signs of discomfort, plus body weight and body temperature deviations) as described previously (17) and listed in Supplementary Figure 2. For each item, mice with no health deficit received a score of 0. If the deficit was mild, they received 0.5 and if it was severe, they received a score of 1. Values for each deficit were summed and divided by all deficits measured to produce an FI score between 0 and 1.

### Echocardiography

Echocardiography was performed on anesthetized mice (isoflurane; induction = 3%; maintenance = 1.5%–2% in oxygen). We used the same mice at 16 and 23 months of age; only mice that were assessed at both time points were included. Mice were immobilized in a supine position on a 37°C platform and chest hair was removed with a depilatory agent. The heart was imaged with a Vevo 2100 echocardiography imaging system (FUJIFILM VisualSonics Inc., Toronto, Ontario, Canada). ECG signals were monitored with limb electrodes to determine heart rate. M-mode echocardiography was used to derive functional and structural parameters from the short axis view: interventricular septum thickness in diastole (IVSd) and systole (IVSs), left ventricular internal diameter in diastole (LVIDd) and systole (LVIDs), left ventricular posterior wall thickness in diastole (LVPWd) and systole (LVPWs), left ventricular (LV) mass, fractional shortening, and ejection fraction. We used pulse-wave Doppler in the apical, 4-chamber view to assess diastolic function. Maximal transmitral inflow velocities were recorded at the mitral orifice, parallel to the direction of blood flow to assess E/A ratios (ratios of peak early-to-late velocities).

### Sample Collection and Analyses

Mice were weighed and anesthetized with sodium pentobarbital (200 mg/kg, IP). Hearts were removed and the ventricles were isolated and snap-frozen in liquid nitrogen. Animals were visually inspected for overt pathology including tumors. Although 5 male and 5 female mice appeared to have various tumors (eg, liver, stomach, or lung), there was no correlation with frailty scores or cytokines and tumor incidence. Ventricular collagen content was measured with a hydroxyproline assay kit (Sigma-Aldrich, St. Louis, MO) following manufacturer’s instructions. Blood samples were obtained either from the facial vein under anesthesia for echocardiography or from the aorta at the end of the study. Blood was spun (4°C; 9391 G) and the supernatant (serum) was collected and stored at −20°C. A mouse 23-plex cytokine assay (Bio-Rad, Mississauga, Ontario, Canada) was used to measure serum concentrations of 23 cytokines (Supplementary Table 1). Assay results were read with a Bio-Plex MAGPIX Multiplex Reader (Bio-Rad, Mississauga, Ontario, Canada). If cytokine concentrations were below the level of detection, values were replaced with the lower limit of detection/2 as described previously (19).

### Statistics

Data are expressed as the mean ± *SEM*. The effects of age and sex on echocardiography parameters and cytokine levels were compared with 2-way repeated-measures ANOVA or mixed effects models as appropriate, with age and sex as main factors. Sidak’s multiple comparisons post hoc test was used. For individual deficits, *t* tests adjusted for multiple comparisons using the 2-stage step-up false discovery rate method were used. Relationships between cardiac outcomes and either frailty or serum cytokines were assessed with Pearson’s *r* test for correlation and simple linear regression was used to plot best-fit curves. For significant correlations, we used multivariable regression and calculated semipartial correlations to assess the separate contributions of age and frailty (or age and serum cytokines). We used SigmaPlot software (v15.0, Systat Software Inc., San Jose, CA), GraphPad Prism (Version 8.2.1, La Jolla, CA), and SPSS software (v21.0, Chicago, IL) for data analysis. Data were graphically displayed with SigmaPlot or Prism. Significance levels less than .05 were considered statistically significant.

## Results

### FI Scores Increased With Age

Prior to echocardiography, we assessed frailty in each mouse. We found that FI scores increased between 16 and 23 months in both male and female mice (Supplementary Figure 1). To determine whether these scores were dominated by deficits in any one specific category, we examined the individual deficits used to create these FI scores. As shown in Supplementary Figure 2 for 16-month-old mice, FI scores reflected deficit accumulation in virtually all systems evaluated. A diverse set of deficits also was seen in these mice as they aged (Supplementary Figure 3). This indicates that FI scores arise from accumulation of many small deficits across a wide range of different bodily systems.

### Impact of Age and Frailty on Heart Function

**Figure 1.**
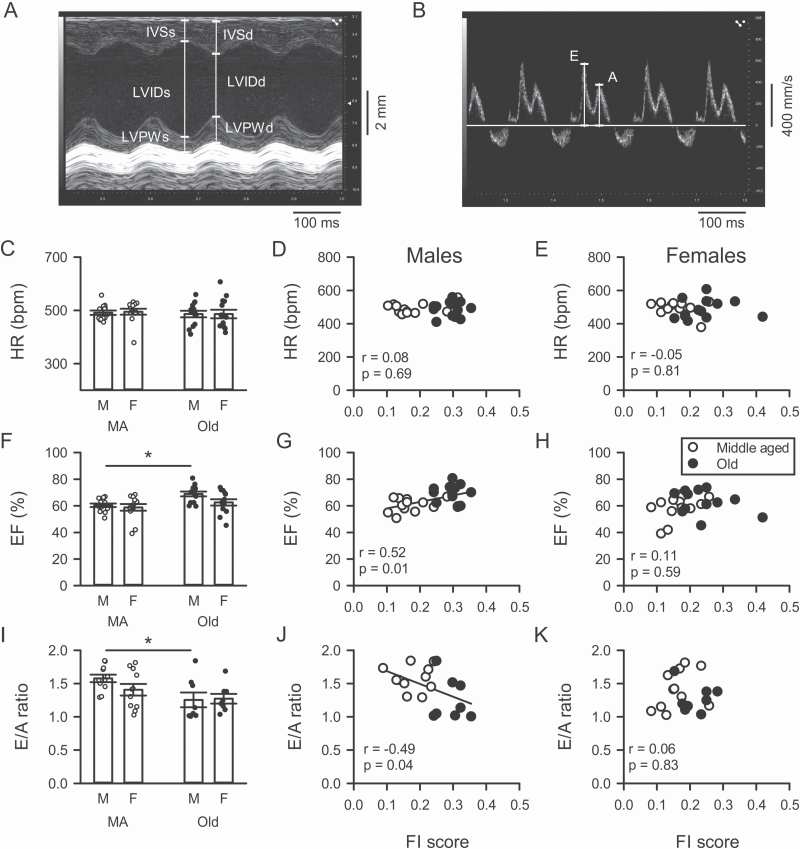
Age-associated changes in systolic and diastolic function are more prominent in male hearts and are graded by the degree of frailty in males but not in females. (**A**) Representative M-mode echocardiography recording from an old male mouse. Parameters of interest measured in this example are illustrated. (**B**) Example of a pulse-wave Doppler recording from an old male mouse. The velocities of mitral valve blood flow, both early (E wave) and later (A wave) are indicated. (**C**) There were no effects of either age or sex on heart rate (HR) measured with echocardiography in this study. (**D**, **E**) There was no relationship between HR and frailty index (FI) scores regardless of sex. (**F**) Ejection fraction (EF) increased with age in males but not females. (**G**, **H**) The increase in EF was closely graded by the levels of frailty in males but this relationship did not exist in females. (**I**) E/A ratios declined with age in males only. (**J**, **K**) The age-related decline in E/A ratio was graded by frailty in males but not in females. Age and sex effects were assessed with 2-way repeated-measures ANOVA or a mixed effects model as appropriate, with Sidak’s multiple comparisons post hoc tests as described in the Method. Correlations were evaluated with a Pearson’s *r* test. The * denotes significant effect of age (*p* < .05). Values of *n* = 13 mice per group for HR and EF; values for the E/A ratios were 11 middle-aged (MA) males, 11 MA females, 8 older males, and 8 older females.

Initial experiments used echocardiography to quantify late-life changes in heart function in mice of both sexes. Figure 1A shows a representative M-mode echocardiography image from a 23-month-old male mouse and Figure 1B shows a pulse-wave Doppler recording from the same animal. Parameters measured in this study are illustrated on the recordings. Figure 1C shows there was no difference in mean heart rates between middle-aged (16 months) and older (23 months) male and female mice. However, there was considerable heterogeneity in values for heart rate between mice of the same ages in both sexes (Figure 1C). We evaluated whether this variability was related to the level of frailty of individual mice. When we plotted heart rate as a function of each animal’s FI score in both groups (Figure 1D and E), we found no relationship between heart rate and frailty scores.

We then assessed age-related changes in contractile function by comparing measures of systolic function (ejection fraction, fractional shortening) in all 4 groups. Figure 1F shows that, on average, ejection fraction increased with age in males but not females. The same pattern was seen with fractional shortening (Supplementary Table 2). As illustrated in Figure 1F, there was substantial variability in all groups, so mice of similar chronological ages could have very different ejection fractions, indicating that factors other than age may affect cardiac contraction. To determine how ejection fraction was affected by frailty, ejection fraction was plotted as a function of each animal’s FI score and fit with linear regression (Figure 1G and H). This analysis showed that ejection fraction was strongly correlated with frailty in male mice (*p* = .01; Figure 1G) but not in female mice (*p* = .58; Figure 1H). As both age and frailty were related to the increase in ejection fraction in males, we used multivariable regression and calculated semipartial correlations to assess the separate contributions of age and frailty. Semipartial correlations showed that both frailty (*r* = 0.52) and age (*r* = 0.61) contributed to the increase in ejection fraction. This shows that age-related changes in ejection fraction in male hearts were graded by the overall health of the animal, as quantified with an FI score. Fractional shortening also was positively correlated with FI scores in males only (Supplementary Table 3); semipartial correlations showed that both frailty (*r* = 0.52) and age (*r* = 0.62) contributed.

To determine how age and frailty affected the ability of the heart to relax (diastolic function), we used pulse-wave Doppler echocardiography (Figure 1B) to measure the E/A ratio (ratio of early [E wave] to late [A wave] ventricular filling). Mean data show that E/A ratios declined with age in males only (Figure 1I). This decrease in E/A ratio, indicative of reduced early ventricular filling and increased late filling, indicates that ventricular relaxation was impaired in older male mice. Still, there was substantial variability in E/A ratios, so individual mice of the same age had very different ratios (Figure 1I). This suggests that factors other than age might account for that variance. Linear regression demonstrated that E/A ratios in males were closely graded by FI score, such that mice with the lowest E/A ratios were the frailest (Figure 1J). By contrast, there was no clear relationship between frailty and E/A ratios in females (Figure 1K). Semipartial correlations revealed that both frailty (*r* = −0.49) and age (*r* = −0.63) contributed to this decline in E/A ratios in males. These findings demonstrate that diastolic function was impaired in older frail male mice.

### Impact of Age and Frailty on Heart Structure

**Figure 2.**
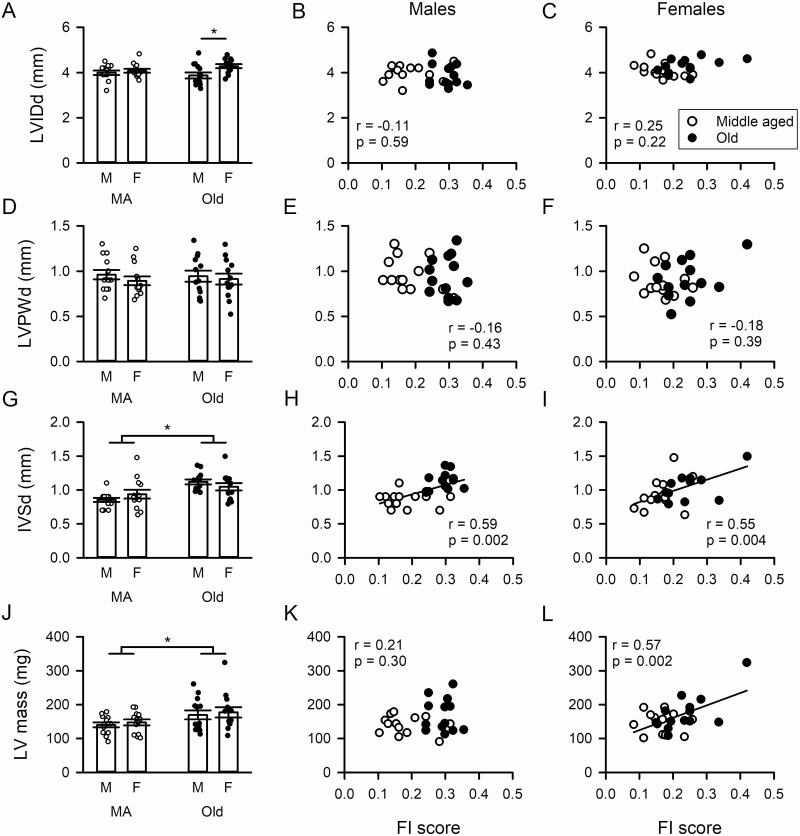
Age-related cardiac remodeling differs between the sexes and is graded by frailty in a sex-specific fashion. (**A**) Left ventricular internal diameter in diastole (LVIDd) was unchanged by age but was higher in older females than older males. (**B**, **C**) LVIDd was not related to frailty scores in either males or females. (**D**–**F**) There was no effect of age, sex, or frailty on left ventricular posterior wall in diastole (LVPWd). (**G**) There was a significant effect of age on intraventricular septum in diastole (IVSd). (**H**, **I**) The age-associated increase in IVSd thickness was closely graded by frailty index (FI) scores in males and females. (**J**) There was a significant increase in left ventricular (LV) mass with age. (**K**, **L**) The age-associated increase in LV mass was graded by the level of frailty in females but this pattern was not observed in males. Data were analyzed with 2-way repeated-measures ANOVA with age and sex as main factors, using Sidak’s multiple comparisons post hoc tests as described in the Method. Correlations with frailty were accomplished with a Pearson’s *r* test. The * denotes significant effect of age or sex (*p* < .05). Values of *n* = 13 mice per group. MA; middle-aged.

M-mode echocardiography can also provide information about the influence of age and frailty on LV structure. Therefore, we next evaluated effects of age on LVIDd (Figure 2A) and LVIDs (Supplementary Table 2). There were few age-related changes in LV internal diameter, although LVIDd was modestly larger in old females than old males, indicative of LV dilation in this group (Figure 2A). Furthermore, there were no correlations between LVIDd (Figure 2B and C) or LVIDs (Supplementary Table 3) and FI score in either sex. Age had no impact on LVPWd (Figure 2D) or LVPWs (Supplementary Table 2) and there was no correlation between these parameters and frailty in males or females (Figure 2E and F). By contrast, both IVSd and IVSs increased with age in both sexes (Figure 2G; Supplementary Table 2). Interestingly, these measures of hypertrophy were positively correlated with frailty (*p* < .001) for both sexes (Figure 2H and I; Supplementary Table 3). Semipartial correlations showed that frailty alone contributed to larger IVSd (*r* = 0.55) and IVSs (*r* = 0.49) in females while age alone contributed to the increase in IVSd (*r* = 0.76) and IVSs (*r* = 0.57) in males. These observations demonstrate that septal wall thickness increased with age and frailty.

We also compared LV mass in male and female mice. Results showed that, although LV mass increased with age in both sexes (Figure 2J), it was positively correlated with FI score in females only (Figure 2K and L). Semipartial correlations showed that frailty alone explained the increase in LV mass (*r* = 0.57). We also measured the impact of age on heart weight-to-body weight (HW:BW) ratios in mice of both sexes (Supplementary Figure 4). We found that HW:BW ratios increased with age (Supplementary Figure 4A), consistent with the age-associated increase in LV mass seen with echocardiography (Figure 2J). HW:BW ratios were correlated with FI scores in males (Supplementary Figure 4B) but this relationship was not statistically significant in females (Supplementary Figure 4C, *p* = .13). Semipartial correlations showed that age (*r* = 0.70) and frailty (*r* = 0.74) were responsible for this effect in males. Taken together, these data suggest ventricular hypertrophy increases with age and frailty in both sexes.

### Influence of Age and Frailty on Ventricular Collagen Levels

**Figure 3.**
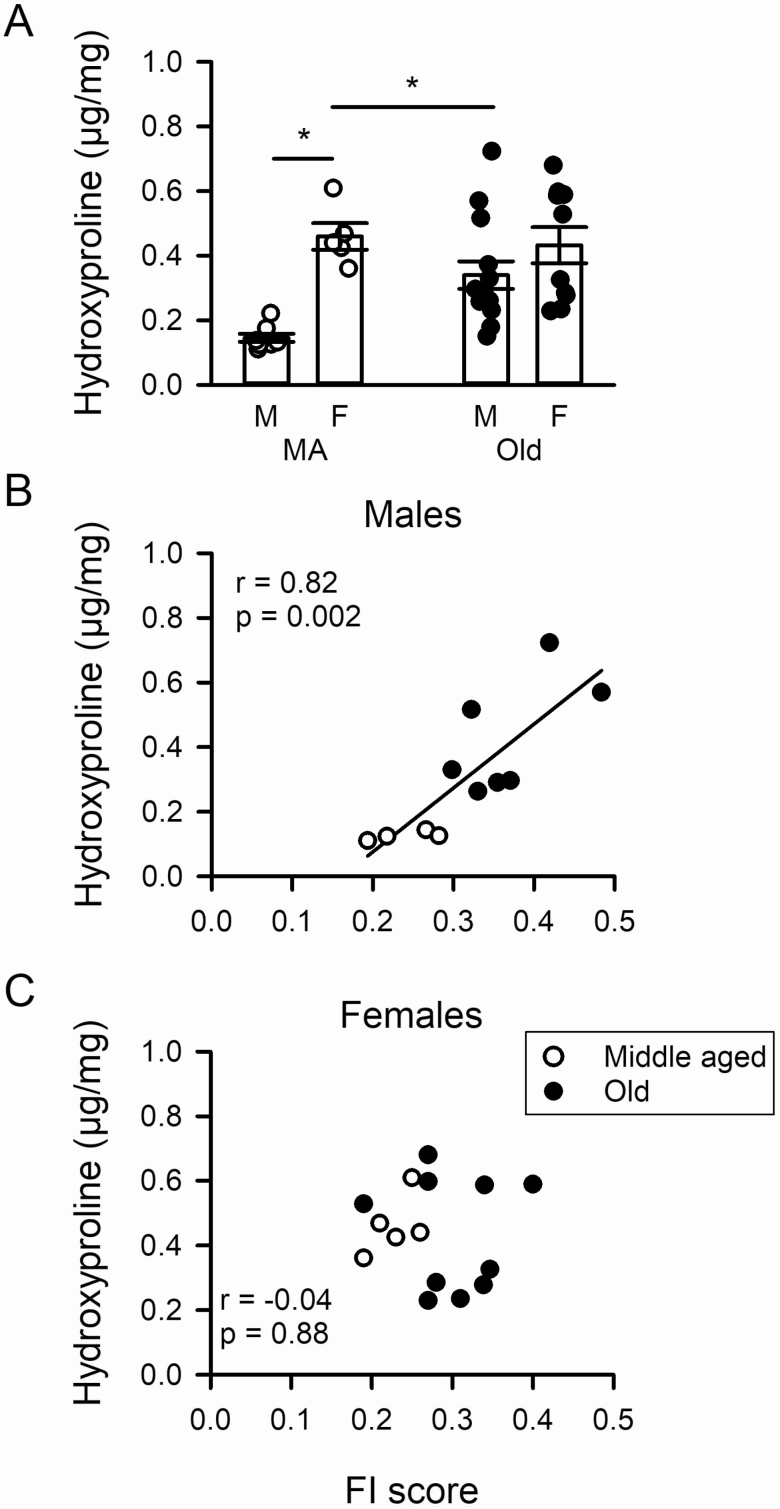
Ventricular collagen levels increase with age in males only and this increase is graded by the level of frailty in males, but not females. (**A**) The levels of hydroxyproline, a major component of collagen, increased with age in males only and were significantly higher in females than males in the younger group. (**B**, **C**) Collagen content was graded by frailty in hearts from males but not females. Data were analyzed with a 2-way ANOVA (Sidak’s multiple comparisons post hoc test) and correlations were assessed with a Pearson’s *r*. The * denotes significant effect of age or sex (*p* < .05). Values of *n* = 4–8 middle-aged males, 5 middle-aged females, 7–14 older males, and 10 older females.

Our Doppler echocardiography data demonstrated that ventricular relaxation was impaired in frail older male mice, which indicates that these animals exhibit diastolic dysfunction. Diastolic dysfunction arises, in part, through ventricular stiffening and fibrosis due to collagen accumulation (24). To investigate this we measured the levels of hydroxyproline, a component of collagen, in the ventricles. We found that total collagen content increased dramatically with age in ventricles from males (Figure 3A). By contrast, collagen levels were significantly higher in middle-aged females than in age-matched males, but collagen did not increase with age in females (Figure 3A). We also found that total collagen content was highly correlated with FI scores in males (Figure 3B). Semipartial correlations showed that frailty (*r* = 0.82) and age (*r* = 0.74) contributed to this increase in collagen in males. By contrast, there was no relationship between collagen and frailty in females (Figure 3C). From these analyses it is apparent that male mice with high FI scores had high levels of ventricular collagen. In contrast, collagen levels were high at all ages examined and no such relationship was observed in females.

### Influence of Age and Frailty on Serum Markers of Inflammation

**Figure 4.**
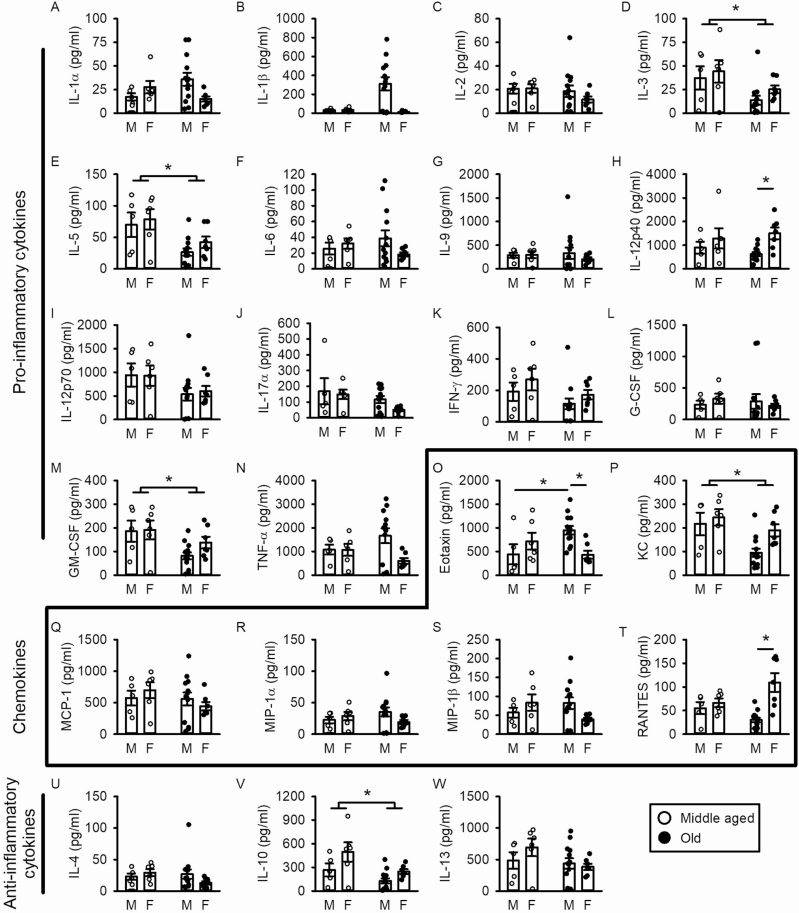
Effects of age and sex on serum levels of inflammatory cytokines and chemokines. (**A–N**) There were few effects of age on serum cytokines and chemokines, although levels of the pro-inflammatory cytokines IL-3, IL-5, and GM-CSF did decline with age in both sexes. There was also a significant effect of sex on IL-12p40 levels, with 23-month-old females having higher levels than age-matched males. **(O–T**) In males only, levels of the chemokine eotaxin increased with age, and at 23 months were significantly higher in males than females. Inversely, levels of RANTES were higher in females than males at 23 months. Levels of KC decreased with age, but there was no effect of age or sex on MCP-1, MIP-1α, or MIP-1β. (**U–W**) Levels of the anti-inflammatory cytokine IL-10 declined with age, and there was a significant effect of sex such that females had higher levels than males. There was no effect of age or sex on serum levels of the other anti-inflammatory cytokines (IL-4 or IL-13). Age and sex effects were assessed with mixed effects models with Sidak’s multiple comparisons post hoc tests as described in the Method. Values of *n* = 5 middle-aged (MA) males, *n* = 13 old males, *n* = 6 MA females, and *n* = 7 old females.

Chronic inflammation is implicated in the development of frailty (11,19) and cardiac fibrosis (25). Therefore, we investigated effects of age and frailty on 23 different cytokines, including “chemotactic cytokines” (chemokines), in serum from aging male and female mice (cytokines listed in Supplementary Table 1). Figure 4 shows mean values for the pro- and anti-inflammatory cytokines and chemokines we measured. There were relatively few changes in cytokines between middle age and later life, although there was marked heterogeneity in values obtained for individual mice (Figure 4). Levels of the pro-inflammatory cytokines IL-3 (Figure 4D), IL-5, (Figure 4E) and granulocyte-macrophage colony stimulating factor (GM-CSF) (Figure 4M) declined with age in both sexes, while older females had higher IL-12p40 levels than age-matched males (Figure 4H). With respect to anti-inflammatory cytokines, there was a significant effect of age and sex on IL-10 levels, with levels declining with age and overall higher in females than males (Figure 4V). Age and sex had no impact on IL-4 or IL-13, which were the other anti-inflammatory cytokines measured (Figure 4U and W). Serum levels of the chemokine keratinocyte chemoattractant (KC) declined with age in both sexes, although females had higher levels than males (Figure 4P). Eotaxin also increased with age in males and was significantly higher in males than females in the oldest mice (Figure 4O). Levels of regulated upon activation, normal T cell expressed and presumably secreted (RANTES) were higher in females than males in the older group (Figure 4T) but there were no effects of age or sex on the other chemokines measured (monocyte chemoattractant protein-1 [MCP-1], macrophage inflammatory protein-1α [MIP-1α] or macrophage inflammatory protein-1ß [MIP-1ß]). These data demonstrate that there are relatively few changes in these markers of inflammation from middle to older ages, although there was variability in levels of cytokines and chemokines in individual mice. Thus, mice of similar chronological ages could have very different inflammatory profiles.

### Links Between Inflammation and Cardiac Function in Aging

**Figure 5.**
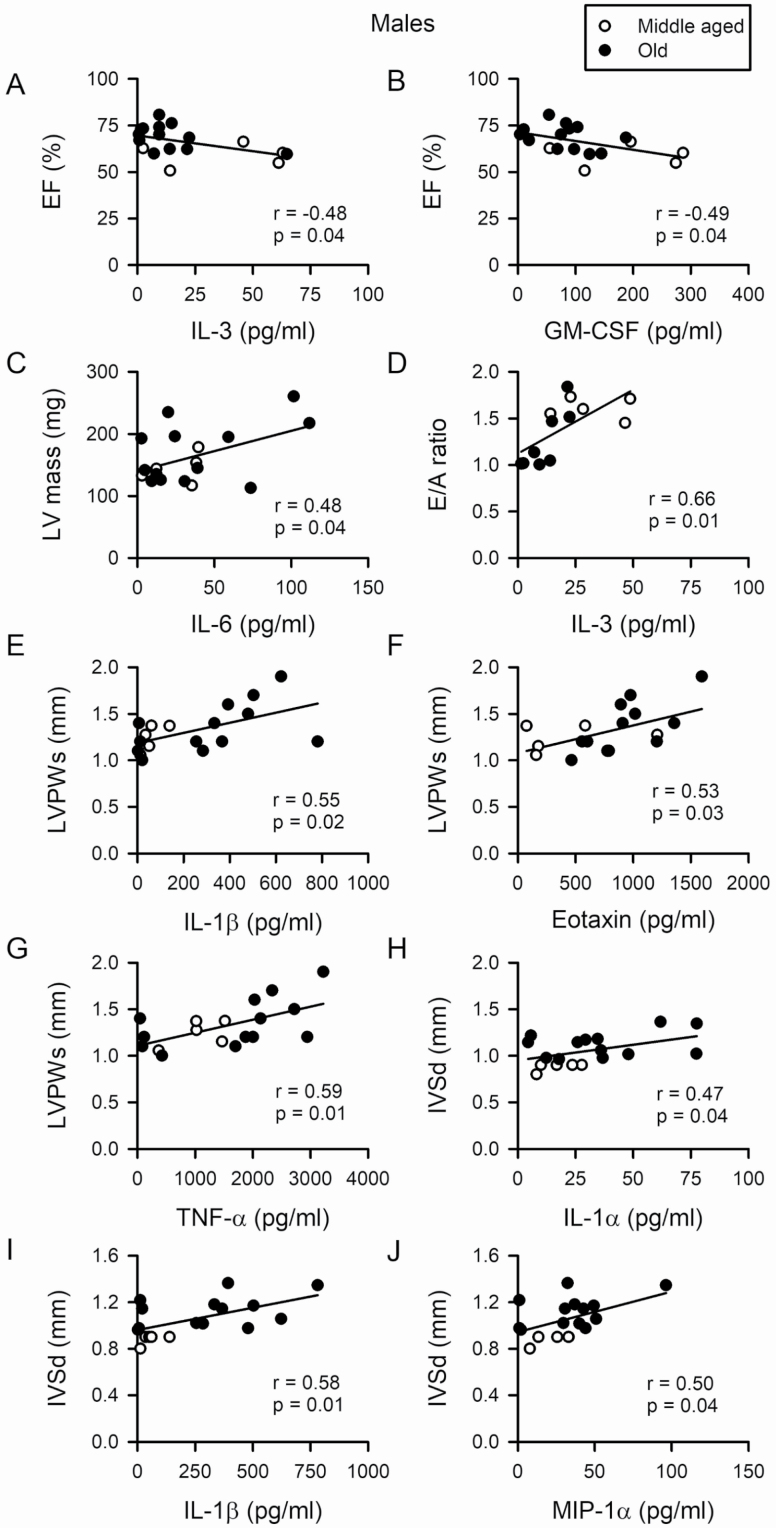
Multiple age-associated changes in myocardial structure and function are closely graded by serum cytokine levels in male mice. (**A**, **B**) Ejection fraction (EF) was inversely proportional to levels of the pro-inflammatory cytokines IL-3 and GM-CSF. (**C**) Left ventricular (LV) mass increased in proportion to levels of IL-6. (**D**) E/A ratios increased as serum levels of IL-3 increased. (**E–G**) An increase in left ventricular posterior wall thickness in systole (LVPWs) was associated with higher levels of IL-1β, eotaxin, and TNF-α. (**H–J**) Intraventricular septum in diastole (IVSd) was positively associated with Il-1α, IL-1β, and MIP-1α. The correlations were conducted with a Pearson’s *r* (*p* < .05). Values of *n* = 5 middle-aged and *n* = 13 old male mice for all measures except E/A ratios where *n* = 5 middle-aged and *n* = 8 old.

As chronic inflammation can promote the development of heart failure (9), we explored associations between inflammation and cardiac outcomes in aging mice. Cytokine levels varied substantially in individual mice, so we used correlations to examine these relationships (Figure 5). Interestingly, while we found clear links between serum markers of inflammation and ventricular remodeling in males (Figure 5), there was no such relationship in females (Supplementary Figure 5). Results in males showed that ejection fraction was inversely proportional to serum levels of the pro-inflammatory cytokines IL-3 (Figure 5A) and GM-CSF (Figure 5B) and semipartial correlations showed that both cytokines (*r* = −0.49) and age (*r* = 0.59) predicted these relationships. E/A ratios were graded by serum levels of IL-3 (Figure 5D) and both cytokines and age contributed to this effect (semipartial correlations were 0.64 and −0.67). Hypertrophy was positively associated with pro-inflammatory cytokines in male mice. LV mass increased as levels of IL-6 increased (Figure 5C); semipartial correlations showed that this relationship was attributable to both cytokines (*r* = 0.48) and age (*r* = 0.26). LVPWs increased as IL-1β, eotaxin, and tumor necrosis factor-α (TNF-α) tumor necrosis factor-α (TNF-α) increased (Figure 5E–G) and this increase was predicted by cytokine levels alone (semipartial correlations were 0.55, 0.53, and 0.59, respectively). IVSd was positively correlated with Il-1α, IL-1β, and MIP-1α (Figure 5H–J), an increase predicted by age alone (semipartial correlations were *r* = 0.69 for each). By contrast, there were no correlations between serum cytokines and any cardiac outcomes measured in female mice. Supplementary Figure 5 shows scatterplots for females for parameters that were correlated with cytokine levels in males. These findings suggest that age-associated changes in ventricular structure and function are most prominent in male mice with high levels of inflammation, a pattern not seen in aging female mice.

## Discussion

We investigated sex-specific differences in effects of frailty and inflammation on adverse changes in the heart in late life. Our data show that aging is associated with adverse changes in ventricular structure and function, as well as enhanced fibrosis, and we report the key finding that these maladaptive changes occur primarily in older males. We demonstrate that many of these alterations are strongly correlated with and graded by frailty and by markers of inflammation in males. In contrast, we found there was little relationship between frailty, inflammation, and cardiac aging in females. These observations show that cardiac aging is correlated with how successfully individual mice age, at least for male animals. Mice that accumulated the most health deficits, and therefore had the highest frailty scores, had more pronounced cardiac aging. These changes were linked to higher levels of inflammation in males but not females. This work also highlights the heterogeneity in the impact of age on the structure and function of the heart, especially the male heart, and shows that both age and frailty contribute to this variance.

Clinical studies suggest that maladaptive changes in the aging heart can differ between the sexes (2). We found that indices of hypertrophy, including LV mass and septal wall thickness, increased with age in mice of both sexes as seen clinically (26). However, measures of systolic function (eg, ejection fraction and fractional shortening) were preserved in aging mice. In fact, these measures showed a small but significant increase with age in males. This is not without precedent. Earlier work suggests that higher ejection fractions in older people arise to compensate for smaller LV end-diastolic volumes (26). We saw no changes in these volumes in mice, so this explanation seems unlikely. A recent study of 2 large clinical cohorts reported a U-shaped relationship between ejection fraction and mortality, with a nadir at an ejection fraction of 60%–65% (27). This increase is not explained by changes in LV volumes, so it is unclear how ejection fraction increases with age and why this increases mortality (27). We recently reported that age, sex, and frailty influence myofilament proteins and their phosphorylation in aging mouse hearts (23). Some of these changes would be expected to enhance contractile function and may compensate for the smaller intracellular calcium transients we observed in ventricular myocytes from older mice (23). Whether these mechanisms help explain increased ejection fraction with age would be interesting to investigate.

Previous studies have reported diastolic dysfunction and elevated fibrosis in aging mice of both sexes (28–32). By contrast, we found E/A ratios declined with age in males only. This may be because we compared older mice to middle-aged mice, while others compared to young adults (30,31). Indeed, we found female mice had high ventricular collagen levels at both ages examined, whereas the males showed an increase between middle age and later life. Thus, cardiac fibrosis may have occurred earlier in females, consistent with clinical findings (2). Investigation of the timing of these changes in mice of both sexes would be of interest. Nonetheless, our findings are important as cardiac fibrosis leads to heart failure with preserved, or even enhanced (33) ejection fraction (HFpEF), the most common form of heart failure in older people (10).

The interanimal heterogeneity in most cardiac outcomes suggests that factors other than chronological age can affect ventricular remodeling. To examine the role of frailty in cardiac aging, we used a validated FI tool based on health deficit accumulation (17). These deficits cover many different systems including the integument, respiratory, digestive, urogenital, musculoskeletal, ocular, auditory, and vestibular systems that are not measures of cardiovascular function per se (34). Interestingly, changes in ejection fraction, E/A ratios, and fibrosis observed in aging males were closely graded by frailty, while there were no links between these measures and frailty in females. Thus, frailty was a strong predictor of cardiac aging in males. Measures of hypertrophy, however, showed a different pattern. Septal wall thickness was positively correlated with frailty in both sexes and LV mass increased in proportion to frailty in females. Together, these findings suggest that poor overall health status predicts ventricular hypertrophy in both sexes. To our knowledge, this is the first study to investigate the influence of frailty on adverse remodeling in in vivo aging mouse hearts. These findings support our previous work in male mice that showed correlations between frailty and ventricular hypertrophy, contractile dysfunction, and atrial fibrosis (35,36). Such associations have also been seen clinically (37). Our findings indicate that frailty is a powerful predictor of adverse ventricular remodeling in aging mice and suggest that frailty creates conditions where diseases like HFpEF can develop and thrive.

Chronic inflammation is an important pathophysiological mechanism in both aging and frailty (11,19). Here, we found large interindividual differences but comparatively few age-associated changes in serum cytokines. Some pro-inflammatory cytokines/chemokines did increase with age in a sex-specific manner including eotaxin, which increased in males, and IL-12p40 and RANTES, which were higher in older females than older males. This is consistent with earlier studies of pro-inflammatory markers in humans. For example, serum levels of eotaxin and IL-12p40 increase with age in people, although this does not differ between the sexes (38,39). Less is known about circulating levels of RANTES clinically, although this chemokine is higher in older female mice when compared to age-matched males (40). We also found that several pro-inflammatory cytokines and chemokines (eg, IL-3, IL-5, GM-CSF, and KC) declined with age in both sexes. This may not be surprising. Age-related declines in cytokines/chemokines including GM-CSF are seen clinically (41).

Earlier work established links between chronic inflammation, aging, and frailty in humans and in mice (42,43). Indeed, a number of inflammatory cytokines/chemokines we investigated here, including IL-1, IL-6, IL-13, TNF-α, interferon-γ, and MCP-1, are elevated in older people and/or mice (19,42–44). These studies typically compare cytokine levels in older individuals with those in young adults (42,44). By contrast, we found no age-associated changes in these cytokines in mice between 16 and 23 months of age. It is possible that levels were already high in the middle-aged mice and that we might have seen an increase if we compared older mice with young adults. In support of this, clinical studies show that serum levels of pro-inflammatory cytokines are highly variable and only higher in older people when compared to levels in much younger individuals (45). This is consistent with our observations in aging mice, where we saw large interindividual variation in cytokine levels in aging mice of both sexes.

We showed that serum levels of the anti-inflammatory cytokine IL-10 declined with age in both sexes. IL-10 is a potent anti-inflammatory cytokine that suppresses the production of many pro-inflammatory cytokines (46). Interestingly, there is evidence that low levels of IL-10 play a role in adverse events linked to aging. For example, IL-10 knockout mice exhibit an accelerated aging phenotype and have been used as a model of frailty (47). Interestingly, these mice also display increased LV mass and diastolic dysfunction (47). Furthermore, high IL-10 levels are associated with longevity and a lower risk of death in humans (46). In addition, aging mice with muscle-specific overexpression of IL-10 show little evidence of muscle inflammation and insulin resistance (48). Thus, the age-related decline in IL-10 levels that we observed in older mice may promote inflammation and adverse changes associated with aging. Interestingly, we found that IL-10 levels were higher in females than males, which may contribute to the protection of female mice against cardiac dysfunction with age.

Chronic inflammation is associated with maladaptive remodeling in diseases such as hypertrophy and heart failure (9,49), so we explored links between inflammation and signs of cardiac aging. We found that, in males, LV mass increased in proportion to serum IL-6 levels. IL-6 is a pro-inflammatory cytokine implicated in the pathogenesis of hypertrophy and heart failure (50). It binds to IL-6 receptors on cardiomyocytes and promotes cardiac hypertrophy in adult male rats (51). Here, we have extended these findings to identify a critical role for IL-6 in age-associated structural remodeling of the male heart, but interestingly not the female heart. We also found that ventricular wall thickness (eg, LVPWs and IVSs) increased in proportion to circulating IL-1α, IL-1β, and TNF-α levels in older male mice. Like IL-6, these are pro-inflammatory cytokines that bind to IL-1 or TNF receptors in cardiomyocytes and promote hypertrophy in adult male rodent hearts (52,53). Our novel observations show that these increases in pro-inflammatory cytokines grade changes in ventricular wall thickness in aging and that this occurs in males only. For several cytokines (eg, IL-6, IL-1α, IL-1β, and TNF-α) we saw associations with adverse cardiac outcomes, even in the absence of an effect of age on these cytokines. This indicates the importance of considering the overall health of mice, not just age, when trying to understand cardiac aging.

We found that ventricular wall thickness (eg, LVPWs and IVSs) increased as levels of the chemokines eotaxin and MIP-1α increased in aging males. Although information is limited, serum levels of these chemokines are correlated with right ventricular hypertrophy in 2 different rodent models of heart failure (54). As chemokines are secondary cytokines induced by pro-inflammatory cytokines including IL-1 and TNF-α (55), the positive correlation between cardiac hypertrophy and eotaxin/MIP-1α in aging males may reflect higher levels of IL-1 and TNF-α. Taken together, our novel results suggest a mechanistic role for pro-inflammatory cytokines and chemokines in ventricular hypertrophy in the aging male heart, an effect that is sex-specific and not seen in females of a similar age.

We showed that many clinically relevant, age-associated changes in heart function are modified by sex and by the overall health of the animal. Still, there are limitations to the work presented here. Our echocardiography studies were performed in anesthetized mice. Although we used the lowest possible amount of anesthetic, anesthesia leads to subphysiological work rates in the heart (56). This could attenuate systolic function and affect our results. Experiments in the presence of a positive inotropic agent such as dobutamine would allow assessment of systolic function under more physiologic conditions. We noted that several mice had tumors, which could affect frailty and cytokine levels. We found no correlation between frailty scores or cytokines and tumor incidence, although the number of mice with tumors was small. We examined relationships between frailty and ventricular structure/function at 2 different ages. It would be informative to perform echocardiography across the life course and track the corresponding frailty scores in individual animals.

It is essential to investigate the influence of age on both male and female animals to address the knowledge gap regarding effects of sex on research outcomes (13). A striking finding in our study is the marked male–female difference in cardiac aging. Although there was a clear relationship between cardiac aging and frailty plus inflammation in males, there was little evidence for this in females. This illustrates the importance of completing studies in both sexes as the use of only one sex would have led to very different conclusions. We found that the impact of age on the heart was heterogeneous and that both frailty and inflammation contributed to this variance in males. This suggests that maladaptive cardiac aging is linked to inflammation and poor overall health in older males. Differences in life span could contribute to these findings if female C57BL/6 mice had a shorter life span than males. However, whether there is a male–female difference in life span in C57BL/6 mice is controversial (57) and we found no sex difference in life span in this strain of mice in our earlier work (16). It is possible that older females are more resilient and may resist some of the adverse effects of frailty and inflammation on the heart. Indeed, the main age-related outcomes seen in female hearts were structural, with aged female hearts displaying increased wall thickness and LV mass, but without the associated changes in function observed in the males. These observations are compatible with the “morbidity–mortality paradox” described clinically, where women are frailer than men at any age, but live longer (21). The reasons for these male–female differences in inflammation and cardiac aging are not well understood, although there is emerging evidence that sex plays a major role in the aging human immune system (58) and that immunosenescence may occur earlier and at faster rates in males than in females (59). A relationship between frailty, inflammation, and cardiac aging might emerge with time in older females.

In summary, our findings demonstrate that older male mice with high levels of frailty and inflammation have more cardiac hypertrophy and dysfunction than those with lower levels of frailty and less inflammation. On the other hand, we found no clear relationship between cardiac remodeling and frailty and/or inflammation in aging females. These data indicate that poor overall health and inflammation are associated with maladaptive cardiac aging in males, but not females. Many cardiovascular diseases, including heart failure, occur in frail, older people. Our findings indicate that frailty and inflammation adversely affect the heart, which may facilitate the development of heart failure in later life. Sex-specific differences in these effects may have important implications for new treatment strategies for diseases like heart failure in aging.

## Supplementary Material

glaa212_suppl_Supplementary_Material
