# Supplementary material for: Maladaptive Changes Associated With Cardiac Aging Are Sex-Specific and Graded by Frailty and Inflammation in C57BL/6 Mice
Source: J Gerontol A Biol Sci Med Sci. 2020 Aug 28;76(2):233–43. doi: 10.1093/gerona/glaa212 (PMC7812442; doi:10.1093/gerona/glaa212)
Supplement: glaa212_suppl_Supplementary_Material [file glaa212_suppl_supplementary_material.pdf]

MALADAPTIVE CHANGES ASSOCIATED WITH CARDIAC AGING ARE SEX-SPECIFIC AND GRADED BY FRAILTY AND INFLAMMATION IN C57BL/6 MICE

Alice E Kane[1,2], Elise S Bisset[3], Stefan Heinze-Milne[3], Kaitlyn M Keller[3], Scott A. Grandy[3,4], Susan E. Howlett[3,5]

[1]Department of Genetics, Harvard Medical School, Boston, MA, USA

[2]Charles Perkins Center, The University of Sydney, Sydney, Australia

[3]Department of Pharmacology, Dalhousie University, Halifax, NS, Canada

[4]School of Health and Human Performance, Dalhousie University, Halifax, NS, Canada

[5]Department of Medicine (Geriatric Medicine), Dalhousie University, Halifax, NS, Canada

Online Only Document Contents:

eTable 1: Cytokines Measured in This Study

eTable 2: Additional Echocardiography Data

eTable 3: Correlations of Echocardiography Data with Frailty Index Scores

eFigure 1. Frailty scores for the mice used in this study.

eFigure 2. Individual deficits and systems evaluated with the mouse clinical frailty index tool in male and female mice at 16 months of age.

eFigure 3. Individual deficits and systems evaluated with the mouse clinical frailty index tool in male and female mice at 23 months of age.

eFigure 4. Heart weight to body weight (HW:BW) ratios for the mice used in this study.

eFigure 5. There Was No Relationship Between Cardiac Functional or Structural Parameters and Serum Cytokine or Chemokine Levels in Female Mice.

eTable 1: CYTOKINES MEASURED IN THIS STUDY

CYTOKINE	ABBREVIATION
Interleukin 1 alpha	IL-1α
Interleukin 1 beta	IL-1β
Interleukin 2	IL-2
Interleukin 3	IL-3
Interleukin 4	IL-4
Interleukin 5	IL-5
Interleukin 6	IL-6
Interleukin 9	IL-9
Interleukin 10	IL-10
Interleukin 12 (p40 subunit)	IL-12(p40)
Interleukin 12 p70	IL-12(p70)
Interleukin 13	IL-13
Interleukin 17 alpha	IL-17α
Granulocyte-colony stimulating factor	G-CSF
Granulocyte-Macrophage Colony Stimulating Factor	GM-CSF
Eotaxin	Eotaxin
Interferon gamma	IFN-γ
Keratinocyte chemoattractant	KC
Monocyte chemoattractant protein-1	MCP-1
Macrophage inflammatory protein-1 alpha	MIP-1α
Macrophage inflammatory protein-1 beta	MIP-1β
Regulated upon Activation, Normal T Cell Expressed and Presumably Secreted	RANTES
Tumour Necrosis Factor alpha	TNF-α

eTable 2: ADDITIONAL ECHOCARDIOGRAPHY DATA

	MALE		FEMALE	
PARAMETER[a]	MIDDLE-AGED	OLD	MIDDLE-AGED	OLD
Heart rate (bpm)	491.4 ± 8.1	486.5 ± 12.4	495.1 ± 11.0	486.5 ± 16.1
Ejection Fraction (%)	60.4 ± 1.4	68.9 ± 1.8*	58.8 ± 2.5	62.6 ± 2.4
Fractional Shortening (%)	31.9 ± 0.9	38.4 ± 1.4*	31.1 ± 1.6	33.9 ± 1.7
E/A Ratio	1.57 ± 0.06	1.25 ± 0.11*	1.41 ± 0.09	1.27 ± 0.07
LVIDd (mm)	3.99 ± 0.10	3.87 ± 0.13	4.08 ± 0.09	4.28 ± 0.09#
LVIDs (mm)	2.72 ± 0.09	2.49 ± 0.13	2.82 ± 0.12	2.84 ± 0.11
LVPWd (mm)	0.96 ± 0.05	0.95 ± 0.06	0.89 ± 0.05	0.91 ± 0.06
LVPWs (mm)	1.35 ± 0.07	1.43 ± 0.07	1.28 ± 0.05	1.37 ± 0.06
IVSd (mm)	0.85 ± 0.03	1.12 ± 0.04**	0.94 ± 0.06	1.05 ± 0.06**
IVSs (mm)	1.39 ± 0.04	1.60 ± 0.05**	1.33 ± 0.07	1.50 ± 0.08**
LV mass (mg)	140.3 ± 7.4	169.5 ± 13.5**	147.3 ± 8.8	177.1 ± 15.3**

[a]Numbers represent the mean ± SEM. Sample sizes were 13 male and 13 female mice in each age group, except for hydroxyproline (MA males n=8, old males n=14, MA females n=5, old females n=10) and E/A ratio (MA males n=11, old males n=8, MA females n=11, old females n=8). Differences between groups were assessed with two-way repeated measures ANOVA or a mixed effects model as appropriate with age and sex as main factors and using Sidak's multiple comparisons post-hoc tests as described in the methods. Differences were significant when $p<0.05$. The * denotes a significant effect of age, the # denotes a significant effect of sex and the ** denotes an overall effect of age.

eTable 3: CORRELATIONS OF ECHOCARDIOGRAPHY DATA WITH FRAILTY INDEX SCORES

	MALE		FEMALE	
**PARAMETER[a]**	**r value**	**p value**	**r value**	**p value**
**Heart rate (bpm)**	r=0.08	p=0.69	r=-0.05	p=0.81
**Ejection Fraction (%)**	r=0.52	p=0.01*	r=0.11	p=0.58
**Fractional Shortening (%)**	r=0.52	p=0.01*	r=0.12	p=0.57
**E/A Ratio**	r=-0.49	p=0.04*	r=0.06	p=0.82
**LVIDd (mm)**	r=-0.11	p=0.59	r=0.25	p=0.22
**LVIDs (mm)**	r=-0.23	p=0.26	r=0.06	p=0.76
**LVPWd (mm)**	r=-0.16	p=0.43	r=0.18	p=0.39
**LVPWs (mm)**	r=0.02	p=0.94	r=0.33	p=0.09
**IVSd (mm)**	r=0.59	P=0.002*	r=0.55	p=0.004*
**IVSs (mm)**	r=0.40	p=0.04*	r=0.49	p=0.01*
**LV mass (mg)**	r=0.21	p=0.30	r=0.57	p=0.002*

[a]Sample sizes were 13 for all groups, except for hydroxyproline (MA males n=4, old males n=7, MA females n=5, old females n=10) and E/A ratios (MA males n=11, old males n=8, MA females n=11, old females n=8). Correlations were conducted with a Pearson's r and * denotes p<0.05.

eFigure 1

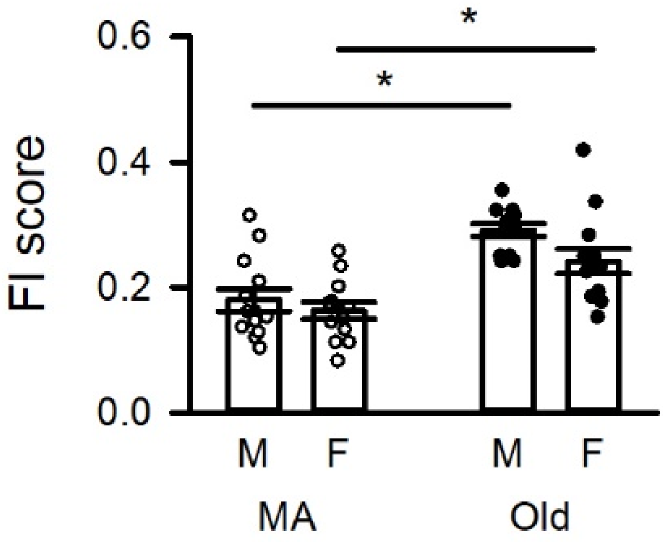

eFigure 2

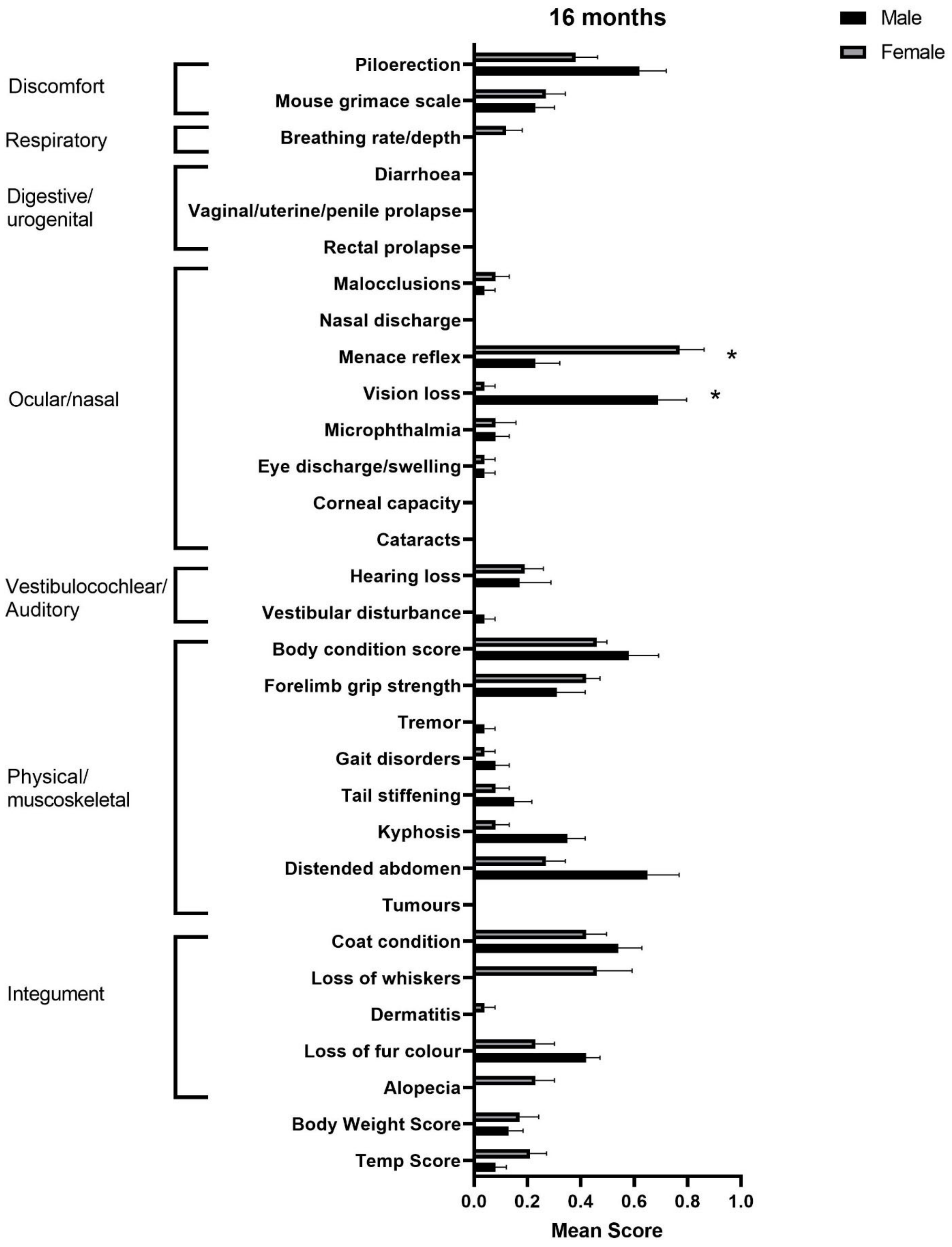
16 months
Male
Female
Discomfort
Respiratory
Digestive/
urogenital
Ocular/nasal
Vestibulocochlear/
Auditory
Physical/
muscoskeletal
Integument
Piloerection
Mouse grimace scale
Breathing rate/depth
Diarrhoea
Vaginal/uterine/penile prolapse
Rectal prolapse
Malocclusions
Nasal discharge
Menace reflex
Vision loss
Microphthalmia
Eye discharge/swelling
Corneal capacity
Cataracts
Hearing loss
Vestibular disturbance
Body condition score
Forelimb grip strength
Tremor
Gait disorders
Tail stiffening
Kyphosis
Distended abdomen
Tumours
Coat condition
Loss of whiskers
Dermatitis
Loss of fur colour
Alopecia
Body Weight Score
Temp Score
*
*
0.0
0.2
0.4
0.6
0.8
1.0
Mean Score

eFigure 3

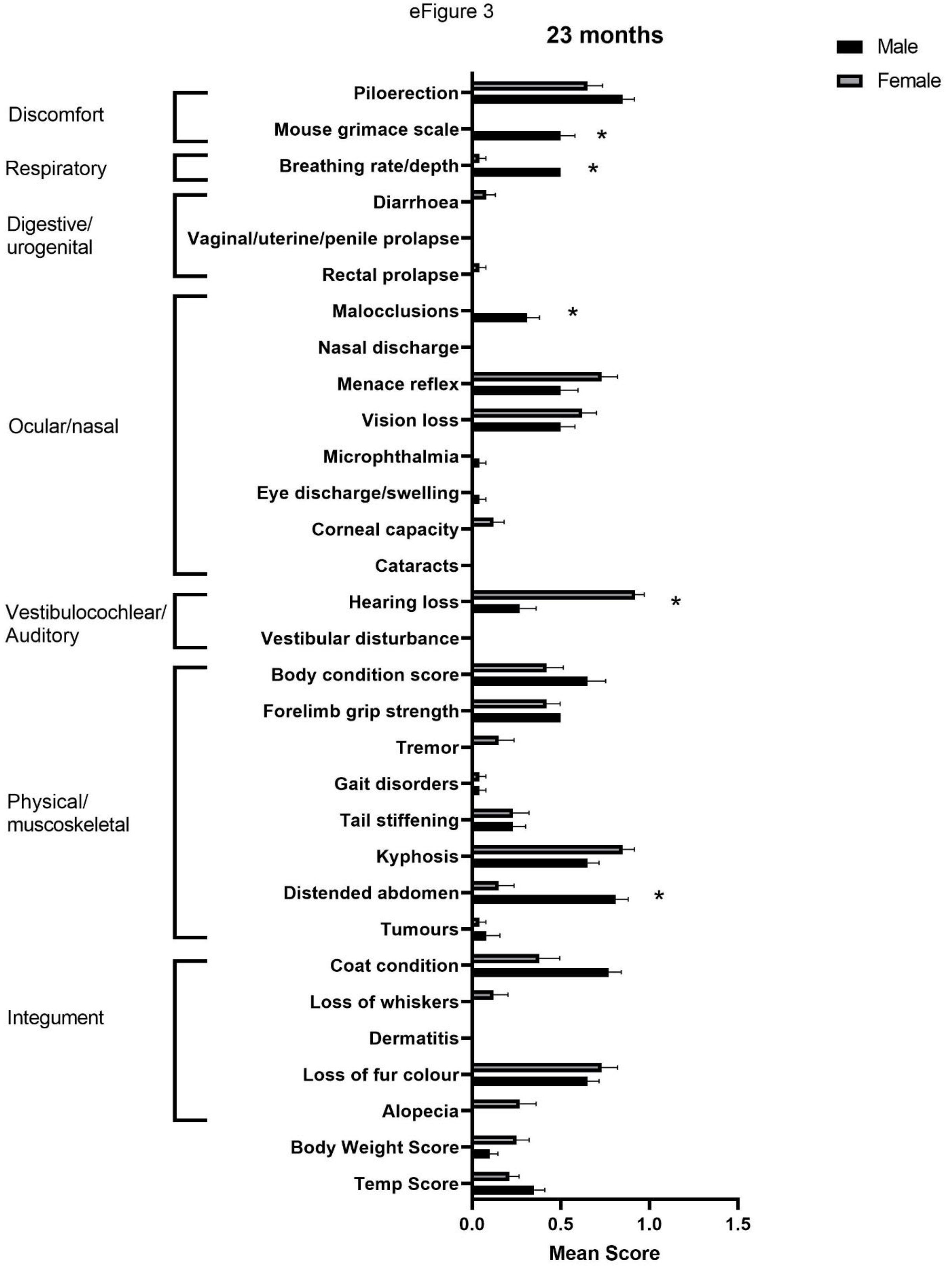
23 months
Male
Female
Discomfort
Respiratory
Digestive/ urogenital
Ocular/nasal
Vestibulocochlear/ Auditory
Physical/ muscoskeletal
Integument
Piloerection
Mouse grimace scale
Breathing rate/depth
Diarrhoea
Vaginal/uterine/penile prolapse
Rectal prolapse
Malocclusions
Nasal discharge
Menace reflex
Vision loss
Microphthalmia
Eye discharge/swelling
Corneal capacity
Cataracts
Hearing loss
Vestibular disturbance
Body condition score
Forelimb grip strength
Tremor
Gait disorders
Tail stiffening
Kyphosis
Distended abdomen
Tumours
Coat condition
Loss of whiskers
Dermatitis
Loss of fur colour
Alopecia
Body Weight Score
Temp Score
*
0.0
0.5
1.0
1.5
Mean Score

eFigure 4

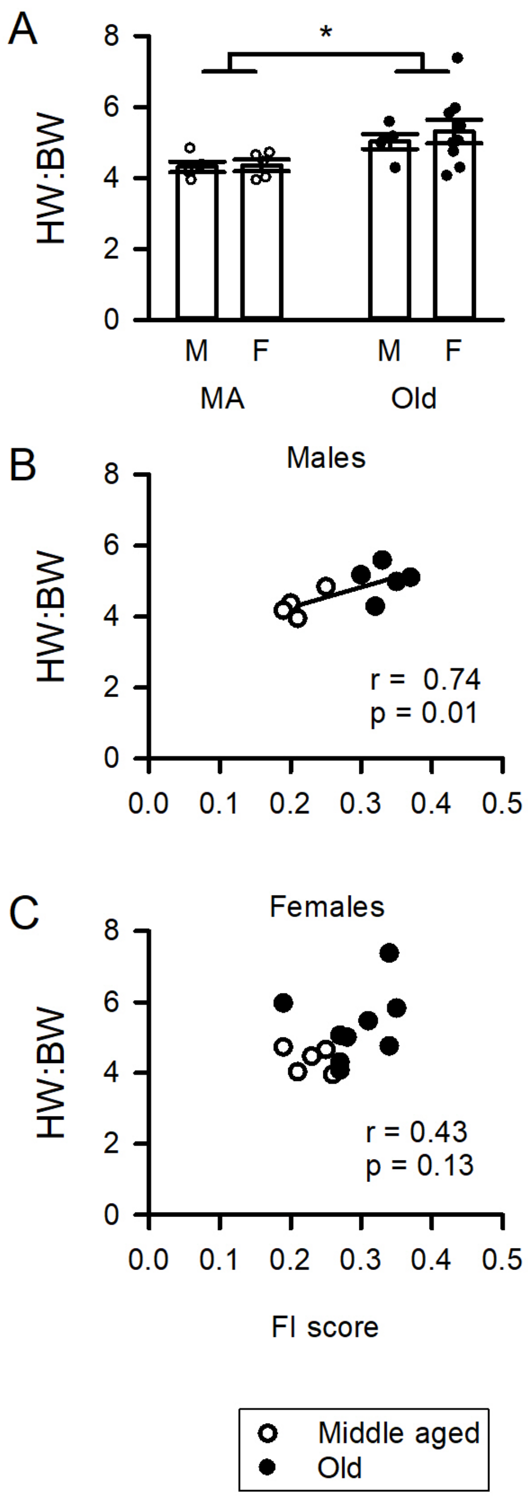
A
HW:BW
8
6
4
2
0
*
M
F
M
F
MA
Old
B
Males
HW:BW
r = 0.74
p = 0.01
0.0
0.1
0.2
0.3
0.4
0.5
C
Females
HW:BW
r = 0.43
p = 0.13
FI score
Middle aged
Old

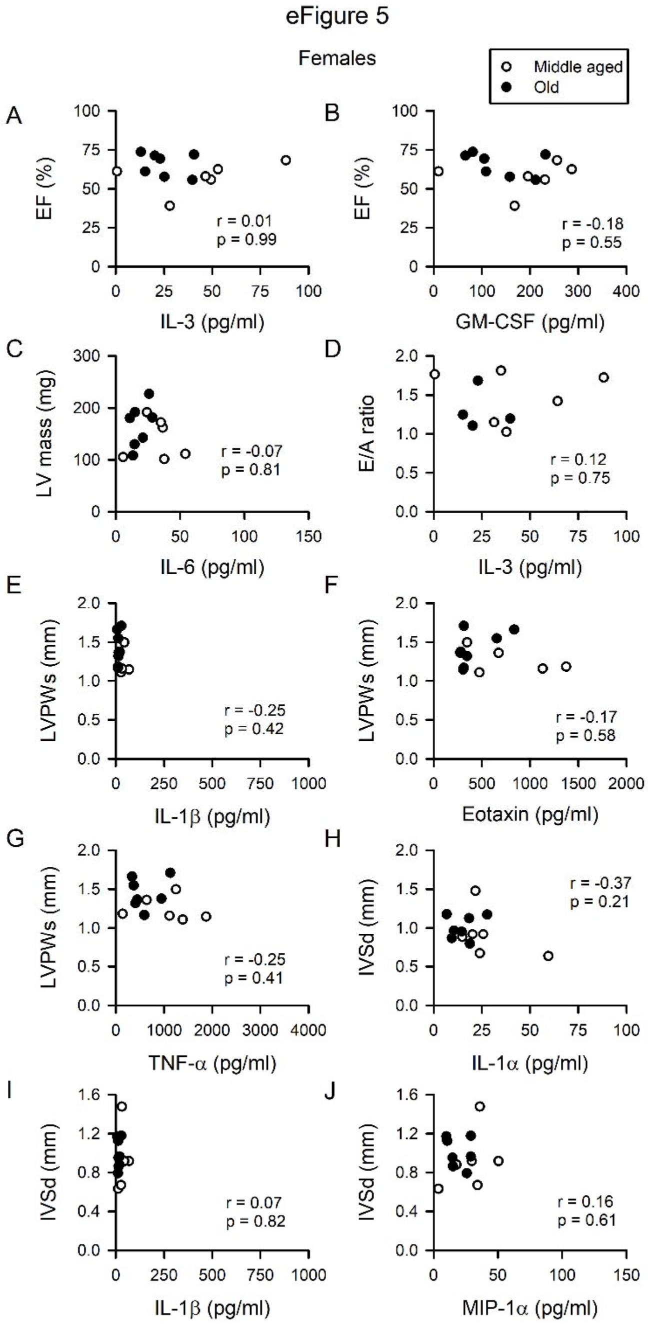
eFigure 5
Females
Middle aged
Old
A
EF (%)
r = 0.01
p = 0.99
IL-3 (pg/ml)
B
EF (%)
r = -0.18
p = 0.55
GM-CSF (pg/ml)
C
LV mass (mg)
r = -0.07
p = 0.81
IL-6 (pg/ml)
D
E/A ratio
r = 0.12
p = 0.75
IL-3 (pg/ml)
E
LVPWs (mm)
r = -0.25
p = 0.42
IL-1β (pg/ml)
F
LVPWs (mm)
r = -0.17
p = 0.58
Eotaxin (pg/ml)
G
LVPWs (mm)
r = -0.25
p = 0.41
TNF-α (pg/ml)
H
IVSd (mm)
r = -0.37
p = 0.21
IL-1α (pg/ml)
I
IVSd (mm)
r = 0.07
p = 0.82
IL-1β (pg/ml)
J
IVSd (mm)
r = 0.16
p = 0.61
MIP-1α (pg/ml)

FIGURE LEGENDS

eFigure 1. Frailty scores for the mice used in this study. Frailty index scores increased with age in both male and female mice. Age and sex effects were assessed with two-way repeated measures ANOVA with Sidak's multiple comparisons post-hoc tests (* denotes $p<0.05$). Values of n=13 middle-aged (MA) and old mice of both sexes.

eFigure 2. Individual deficits and systems evaluated with the mouse clinical frailty index tool in male and female mice at 16 months of age. Health deficits were present across a wide range of different body systems in 16-month-old mice of both sexes. There were few statistically significant differences between the sexes. Differences between the sexes were evaluated with t-tests adjusted for multiple comparisons using the two-stage step-up false discovery rate method (* denotes $p<0.001$). Values of n=13 middle-aged (MA) and old male and female mice.

eFigure 3. Individual deficits and systems evaluated with the mouse clinical frailty index tool in male and female mice at 23 months of age. Health deficits were present across a wide range of different body systems in 23-month-old mice of both sexes. Deficits increased with age but, as seen in the same mice at 16 months, there were few statistically significant differences between the sexes. Differences between the sexes were evaluated with t-test adjusted for multiple comparisons using the two-stage step-up false discovery rate method (* denotes $p<0.001$). Values of n=13 middle-aged (MA) and old male and female mice.

eFigure 4. Heart weight to body weight (HW:BW) ratios for the mice used in this study. A. HW:BW ratios increased with age in both male and female mice. **B.** There was a statistically significant correlation between HW:BW ratios and frailty scores in male mice. **C.** In contrast to males, the correlation between HW:BW ratios was not statistically significant in females. Age and sex effects were assessed with a mixed-effects model with Sidak's multiple comparisons post-hoc tests (* denotes $p<0.05$). The correlations were conducted with a Pearson's r ($p<0.05$). Values of n=5 middle-aged (MA) males and females, and old males and n=9 old females.

eFigure 5. There was no relationship between cardiac functional or structural parameters and serum cytokine or chemokine levels in female mice. There were no correlations between serum cytokines/chemokines and any of the echocardiography parameters measured in this study in female mice. This figure illustrates the scatterplots for the parameters that were correlated with cytokine levels in males. **A-B.** Ejection fraction (EF) was not associated with IL-3 or GM-CSF levels in females. **C-D.** There was no relationship between LV mass and IL-6, or E/A ratio and IL-3. **E-G.** Unlike males, LVPWs was not correlated with levels of IL-1ß, Eotaxin or TNF-α. **H-J.** IVSd was not associated with serum Il-1α, IL-1ß and MIP-1α. The correlations were conducted with a Pearson's r ($p<0.05$). Values of n=13 MA and old male and female mice for all measures except E/A ratios where n=11 MA and n=8 old.
